# Clinical profiles and care of transgender children and adolescents who receive specialized consultations: do individuals who are assigned female at birth differ?

**DOI:** 10.1186/s13034-024-00809-w

**Published:** 2024-11-07

**Authors:** Chystelle Lagrange, Nina Verger, Julie Brunelle, Fanny Poirier, Hugues Pellerin, Nicolas Mendes, Gregor Mamou, Nifoular Forno, Laetitia Martinerie, David Cohen, Agnès Condat

**Affiliations:** 1 Service de Psychiatrie de l’Enfant et de l’Adolescent, APHP.SU, Sorbonne Université, Groupe Hospitalier Pitié-Salpêtrière, Paris, France; 2 Réseau Trajectoire Trans d’Ile de France, APHP, Paris, France; 3 Service d’Endocrinologie Pédiatrique, hôpital Robert Debré, APHP, et Université Paris Cité, Paris, France

**Keywords:** Gender incongruence, Gender dysphoria, Comorbidity, Childhood, Adolescence, Treatment

## Abstract

**Context:**

Clinical data on transgender children and adolescents are scarce, and sample sizes often do not allow for comparisons according to sex assigned at birth. Besides, most gender identity clinics have pointed to a recent switch in favor of an increase in the number of adolescents assigned females at birth (AFAB) over assigned males at birth (AMAB).

**Method:**

We collected data on sociodemographic characteristics, and psychiatric and social vulnerabilities according to sex assigned at birth for all youths who were treated at the French largest gender identity clinic. In addition, management modalities for gender transition were discussed in multidisciplinary concertation meetings.

**Results:**

We collected data from 239 youths [162(68%) AFAB, 74(32%) AMAB, and 3(1%) intersex; mean age = 14.5(± 3.16) years]. The distribution of age at referral was better explained by two clusters (C1: *N* = 61, mean age = 11.3 years, with more AMAB; C2: *N* = 175, mean age = 15.9 years with more AFAB). 215(91%) youths had gender incongruence, with 32% reporting it before puberty. School drop-out, suicidality, depression, and anxiety were common and occurred significantly more often in the AFAB group. 178(74%) youths experienced social transition within the family, and 144(61%) at school [mean age = 15.13(± 2.06) years]. The social transition was more frequent in the AFAB group. Twenty-six (11%) youths received puberty blockers [mean age = 13.87(± 2.31) years], and 105(44%) received gender-affirming hormones [mean age = 16.87(± 1.4) years]. AMABs were more likely to take puberty blockers, and there was no difference in the proportion of AMAB and AFAB taking gender-affirming hormones. Surgical requests (mainly torsoplasty) were very rare.

**Conclusion:**

Age at referral should be considered when exploring gender incongruence. During adolescence, we found that gender incongruence has substantial social and psychological effects, particularly on AFAB youths, possibly explaining their higher referral rates to specialized centers, as in other specialized clinics around the world.

## Background

Recently, there have been significant changes in how we understand and support young people dealing with gender identity challenges. In the latest International Classification of Diseases (ICD-11), the World Health Organization defines gender incongruence as “a marked and persistent incongruence between the gender experienced by an individual and the gender assigned at birth” [1]. While comprehensive prevalence data on gender incongruence among the general population of children and adolescents are not available, targeted school-based studies offer some indications. It appears that 1.3–2.7% of school children self-identify as transgender or gender nonconforming people [2–4]. In health care systems, the prevalence of transgender individuals receiving gender-affirming treatment is much lower and varies according to inclusion rule, year, and definition used. Notably, the prevalence of transgender or gender-diverse Medicare beneficiaries increased by a factor of 4 between 2010 and 2016 [4]. The most recent study in the UK reported a prevalence of 0.03% for gender-affirming treatment. However, the registration rates across different age groups diverged substantially after 2010. By 2018, the highest percentage of individuals who identified as transgender was found in the 16–17-year age group (0.16%) and the 18–29-year age group (0.12%) [5]. In France, although data to evaluate gender incongruence in the general youth population are not available, public health service records reveal that in 2020, 3.3% of the 8952 individuals who received full reimbursement for medical expenses related to “trans identity” were minors [6]. More individuals who are assigned male at birth (AMAB) than individuals who are assigned female at birth (AFAB) are reported, with the notable exception of minors, with more individuals who are AFAB than individuals who are AMAB reported [6].

Gender incongruence often leads to significant psychological and social distress, defined as gender dysphoria in the DSM-5. Individuals with gender incongruence report experiences stemming from discrimination and marginalization that create unique stressors; these stressors, combined with co-occurring mental health concerns, are important to consider during assessment [7]. A recent meta-analysis revealed that the pooled mean age at referral to specialized clinics was 13.2 years [8], emphasizing the critical role of specialists in supporting these often marginalized and suffering adolescents. Indeed, numerous studies indicate that transgender youth are significantly more likely to have mental health issues than their cisgender peers [9]. A recent meta-analysis, for example, revealed a prevalence of 28% for suicidal ideation and 14.8% for suicide attempts in transgender youth [10]. In addition to mood disorders and anxiety, there is also a higher prevalence of autism spectrum disorder (ASD), eating disorders, and substance abuse, highlighting the profound need for care for these individuals [11]. Additionally, it appears that the social characteristics of young people who are transgender or questioning include more minority characteristics [12], and a large number of these individuals have some form of socioeconomic deprivation [5].

Despite the increase in the number of young transgender people seeking support from specialized services, qualitative research shows that they often feel unsatisfied with their care and perceive a lack of understanding from professionals [13]. This dissatisfaction could be attributed to the insufficient specific information available about the young transgender population, especially for distinct subgroups like subgroups according to sex assigned at birth. Epidemiologically, most gender identity clinics have pointed a switch in favor of an increase in the number of young transgender males (AFAB) over young transgender girls (AMAB): In London [14], Toronto [15], Amsterdam [16], Paris [17], Sweden [18], Belgium [19], leading to the development of the Rapid Onset Gender Dysphoria (ROGD) concept [20, 21]. ROGD has been contested by many authors in the field as it suffers social and methodological critiques [22–24]. Notably, these critiques point out the psychopathologizing and stigmatizing premises about being transgender, and the selection and sampling of respondents (mainly parents of trans youth recruited on online platforms harboring trans-antagonistic content). ROGD is best understood as an attempt to circumvent existing research demonstrating the importance of gender affirmation, relying on scientific-sounding language to achieve respectability [22]. Nonetheless, age at the time of seeking care varies across European clinics for individuals who are AMAB and those who are AFAB [14]. The psychological problems and psychiatric comorbidities of minors who are AFAB and AMAB are still debated. Some studies have reported poorer mental health in young individuals who are AFAB, particularly in terms of depression and anxiety [25], self-harm [26], and suicidality [27]. However, other studies have shown the opposite, indicating that individuals who are AMAB might have worse mental health [28, 29]. Studies comparing the transition pathways of these two groups are rare, but data from the large Amsterdam Cohort suggest that transgender female youths may start hormone treatment at an older age and more frequently than transgender male youths [30].

### Aims of the study

Here, we report our experience of 239 minors seeking care in the largest specialized clinics in Paris, France. Given the recent switch towards AFAB adolescents, we aimed (i) to explore age at referral in our sample using admixture analysis, a common clustering method to characterize the nonnormal distribution of continuous variables; (ii) to compare the demographic characteristics, psychiatric comorbidities, and social and medical transition pathways of individuals who were AFAB and AMAB and assessed over 10 years. By clearly outlining the differences between these subgroups, our goal is to uncover their specific needs and thus improve the care we provide.

## Methods

### Study design and participant recruitment

The study included data from all 239 children and adolescents who visited the specialized Gender Identity Clinic (GIC) of La Pitié-Salpêtrière Hospital from June 2012 to March 2022. This clinic, which opened in 2013, is part of the largest care network in France (Trajectoire Jeunes Trans) and provides medical care and support for children and adolescents (under 18 years of age) who question their gender identity and their families. The GIC accepts patients referred by doctors, support groups, or those who approach the clinic independently. The care network is composed of a multidisciplinary healthcare team that includes psychologists, occupational therapists, child and adolescent psychiatrists, endocrinologists, surgeons, pediatricians, biologists, ethicists, and lawyers. Usual care may involve providing psychological counseling for children and their families, managing gender dysphoria and co-occurring psychiatric problems, or providing referrals for medical interventions such as hormonal therapy or gender-affirming surgery. Hormonal interventions include treatment with gonadotropin-releasing hormone (GnRH) agonists to halt development during puberty that does not align with a patient’s gender identity and gender-affirming hormones (testosterone or estradiol) to promote the development of congruent secondary sexual characteristics. The GIC follows the international guidelines published by the World Professional Association for Transgender Health [31] and the Endocrine Society [32]. Additionally, any decision to start hormonal therapy or surgery is collectively discussed in mandatory medical meetings within our network to prevent isolated decision-making (called “RCP, Réunion de Concertation Pluri-disciplinaire”). These meetings, which also involve support group representatives, are open to families and adolescents who wish to attend. All care options, including these meetings, are only based on a patient’s needs, with the patient’s preferences remaining central to the care provided by the GIC.

Our cohort was composed of all patients who were treated at the GIC up to March 2022. The only inclusion criterion was, therefore, having visited La Pitié-Salpêtrière GIC at least once before turning 18 years old between June 2012 and March 2022. There was no minimum age (the youngest child was 3 years old). People with all kinds of gender identity questioning were included. To maintain clarity, we use the terms assigned male at birth (AMAB) and assigned female at birth (AFAB). We included data from people with or without gender dysphoria/incongruence symptoms as described in the ICD-11 criteria [1].

### Variables

To assess sociodemographic or clinical differences between the AMAB and AFAB populations in our cohort, we extracted data on the following variables from patients’ medical charts and the computerized database dedicated to the GIC: (1) sociodemographic characteristics, (2) psychiatric history and current diagnosis, (3) social transition status, and (4) gender-affirming treatment decisions after the RCP.

Sociodemographic characteristics included age at intake, sex assigned at birth (AFAB, AMAB or intersex), gender expressed (trans male, trans female, nonbinary or in questioning), and family situation (living with both parents, living with one parent, separated parents, or living in a children’s social care institution). We assessed socioeconomic status using the occupation of both parents, referencing the *Institut national de la statistique et des études économiques* [33] classification and normalized it using a Likert scale ranging from 1 to 3 (1: low status, 2: middle status, and 3: high status). We also recorded school situations (regular education, specialized education, out of school) and sexual orientation (heterosexual, homosexual, bisexual, pansexual, or not communicated).

For psychiatric history and current diagnoses, we determined the presence or absence of gender incongruence, as defined by the ICD-11 criteria, and identified whether this incongruence was present before puberty. We also determined whether gender dysphoria was present using the Zucker scale [34]. Psychiatric history information was obtained from medical records or interviews (and confirmed by parents or educational institutions), including prior psychiatric hospitalization, experiences of bullying, interruption in schooling, the presence of suicidal thoughts, and suicide attempts. Additionally, we systematically evaluated the prevalence of psychiatric comorbidities according to the DSM-5 criteria during clinical interviews using the Mini International Neuropsychiatric Interview for Children and Adolescents [35]. Considering the department’s expertise and the notable occurrence of educational issues in the study sample, we also systematically evaluated the presence of learning disabilities.

We also investigated aspects of social transition, such as the affirmation of gender at school, within the family, and among peers, as well as any changes in legal identification (name and gender marker). Notably, according to French law, a change of name is possible at local town halls without a minimum age requirement, with the agreement of both parents. Gender changes on official documents are possible from the age of 18 years (or 16 years if the teenager is legally emancipated). Finally, we investigated whether the patients had pursued medical interventions, including hormone treatments (GnRH agonists and/or gender-affirming hormones), or surgical procedures.

### Statistical analysis

Statistical analyses were conducted with R 4.0.3 software using two-tailed tests with a significance level set at 5%. We first described the overall sample, regardless of the sex assigned at birth. The distribution of the qualitative variables is shown by the number and percentage of occurrences of each modality. The distribution of the quantitative variables is described by the mean and standard deviation or by the median and the interquartile range. Quantitative variables were compared with Welch’s *t*-tests or Wilcoxon rank sum tests, depending on whether the conditions for test validity were met. Qualitative variables were compared with chi-square tests or Fisher’s exact tests, depending on whether the conditions for test validity were met. For expressed gender, which has more than two modalities, quantitative variables were compared with ANOVA or the Kruskal‒Wallis test. Pairwise comparisons were performed for variables that were significant in the overall test. Due to the exploratory nature of the study, p values for two-to-one comparisons are presented without multiplicity adjustment [36]. Given the lack of normal distribution of age at referral, we conducted an admixture analysis to model its distribution.

## Results

### Sociodemographic characteristics

**Fig. 1:**
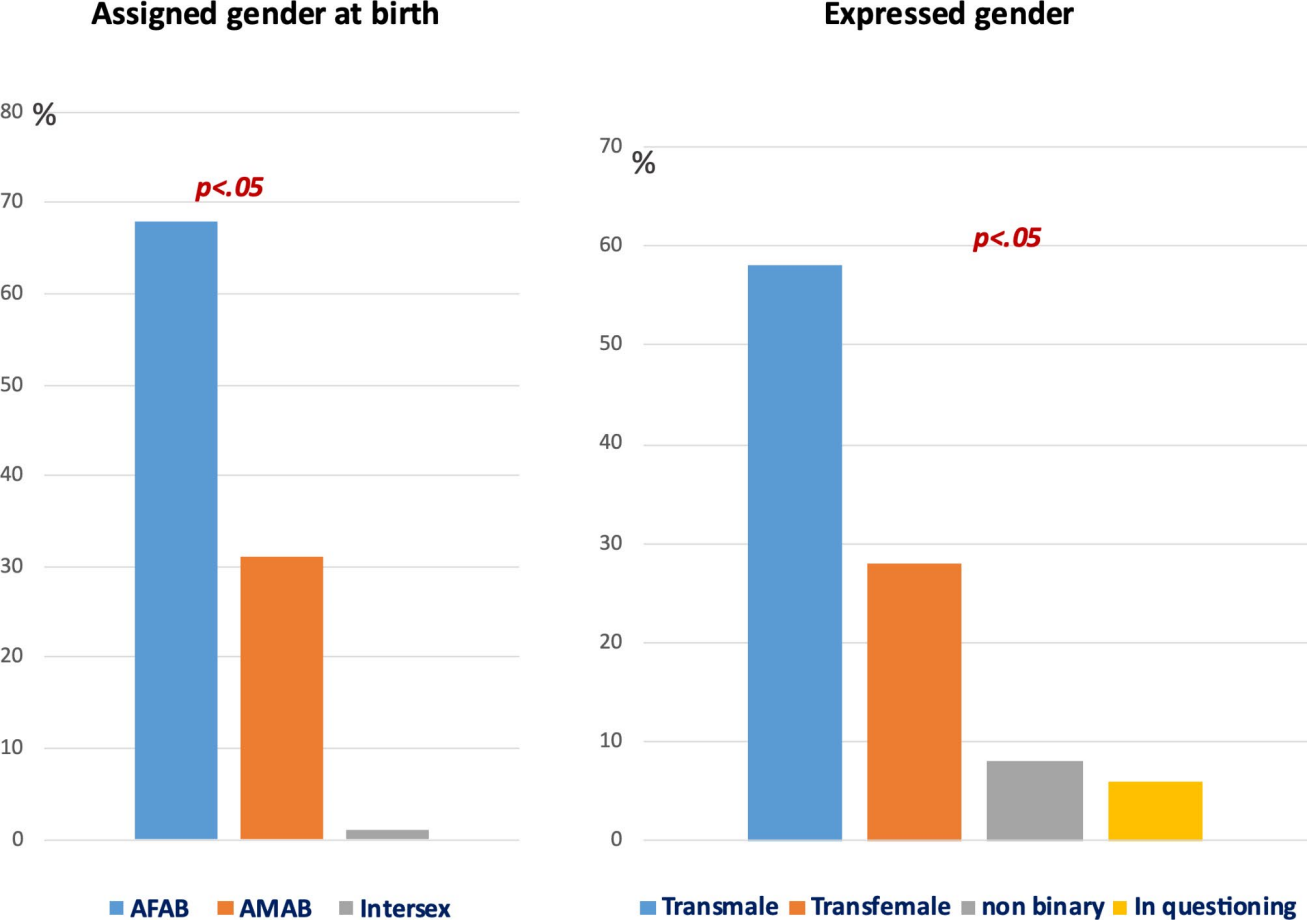
Assigned gender at birth and expressed gender for the children and adolescents who were assessed for gender incongruence between 2012 and 2022

Table 1 summarizes the sociodemographic data for the 239 children and adolescents who visited the GIC according to their expressed gender. In total, we included 162 (68%) individuals who were assigned female at birth (AFAB), 74 (32%) who were assigned male at birth (AMAB), and 3 (1%) who were intersex with differences in sex development. Comparisons between the AFAB and AMAB subgroups were made using a total sample of 236 children and adolescents. As Fig. 1 shows, there were notably more youths who were AFAB than youths who were AMAB. We found that the age at intake was significantly lower for the AMAB group. There was no significant difference in family structure, socioeconomic status, or education between the two groups. Most youths from both groups lived with both parents, belonged to middle or high socioeconomic classes, and attended regular school.

**Table 1:** Sociodemographic characteristics of individuals who were AFAB and AMAB

Variables	AFAB (*n* = 162)	AMAB (*n* = 74)	Total (*n* = 239)*	Test	*p* value
Age at on set					
Mean (SD) Median [q1; q3]	15.11 (2.5) 15.7 [14.4; 16.5]	13.13 (3.99) 14.96 [10.3;16.4]	14.5 (3.16) 15.5 [13.7;16.5]	W	**0.004**
Family situation				Chi2	0.424
Lives w. both parents	100 (61.7%)	39 (52.7%)	142 (59.4%)		
Lives w. one parent	42 (25.9%)	24 (32.4%)	66 (27.6%)		
Separated parents	9 (5.6%)	5 (6.8%)	14 (5.9%)		
In care of social services	8 (4.9%)	5 (6.8%)	13 (5.4%)		
Others	3 (1.9%)	1 (1.4%)	4 (1.8%)		
Socioeconomic status				Chi2	0.752
1. Low 2. Middle 3. High	33 (20.4%) 64 (39.5%) 65 (40.1%)	12 (16.2%) 31 (41.9%) 31 (41.9%)	45 (18.8%) 95 (41%) 96 (40.2%)		
Education				Chi2	0.365
Regular Specialized Out of school	103 (63.6%) 12 (7.4%) 47 (29.3%)	54 (73%) 4 (5.4%) 16 (21.6%)	159 (66.5%) 17 (7.1%) 63 (26.4%)		
Sexual orientation					
Heterosexual Homosexual Bisexual Pansexual Not communicated	24 (10.1%) 27 (11.4%) 4 (1.7%) 10 (4.2%) 174 (73.4%)				
Gender incongruence diagnosis, ICD-11	155 (95.7%)	60 (81.1%)	215 (91.1%)	Chi2	**< 0.001**
Gender incongruence started before puberty	41 (25.3%)	34 (45.9%)	75 (32%)	Chi2	**0.002**

*n = 239 refers to *n* = 236 individuals who were AFAB and AMAB plus 3 intersex individuals who were not included in the statistical comparisons

AFAB assigned female at birth, AMAB assigned male at birth, w. with, in bold: *p* <  0.05

Information regarding the sex assigned at birth of the patients treated at the GIC is provided in Fig. 1. The number of patients with gender incongruence according to the ICD-11 criteria was significantly greater in the AFAB group (Table 1). As expected, there were significantly more trans males (*n* = 138) than trans females (*n* = 68). In addition, there were a few nonbinary youths (*n* = 19) and a few youths who questioned their gender (*n* = 14). However, for individuals with gender incongruence before puberty (*n* = 75, 32%), the AMAB group had a significantly greater proportion (*n* = 34, 45.9%) than did the AFAB group (*n* = 41, 25.3%, *p* = 0.002). Only 65 youths expressed a view of their sexual orientation: more than 2/3 had no specific orientation yet; 24 (10.1%) were heterosexual, 27 (11.4%) were homosexual, 10 (4.2%) were pansexual, and 4 (1.7%) were bisexual. Due to the limited information on sexual orientation for most youths, we did not perform statistical analysis.

**Fig. 2:**
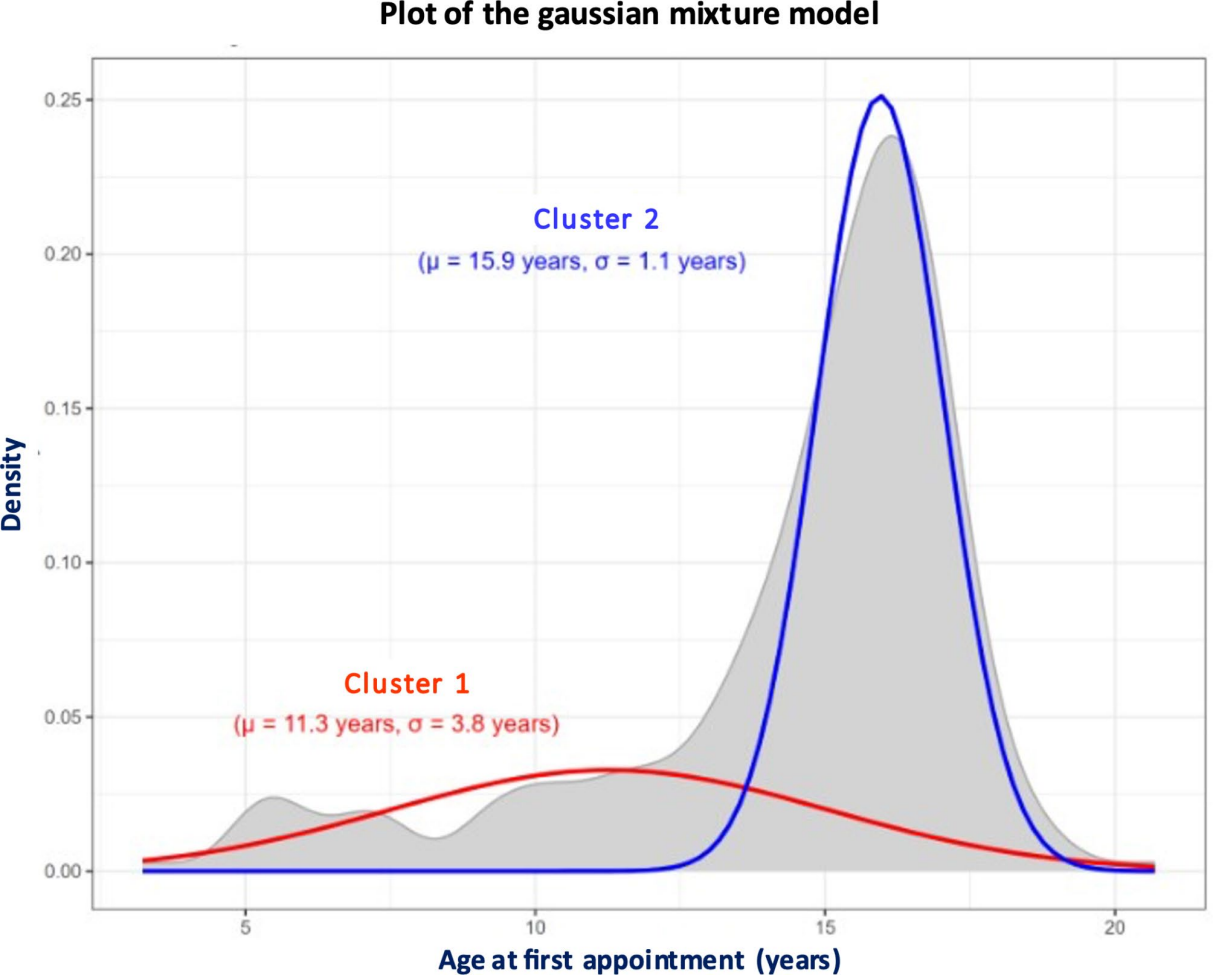
Admixture analysis of the distribution of age at referral for the children and adolescents who were assessed for gender incongruence between 2012 and 2022

Given the lack of normal distribution of age at referral, we conducted an admixture analysis to model its distribution. Real distribution and the best model are shown in Fig. 2. Admixture analysis suggests that the distribution of age at referral was better explained by two clusters. Cluster 1 includes 61 individuals (30(49.2%) AFAB and 31(50.8%) AMAB), with a mean age equal to 11.3 (± 3.8) years, whereas Cluster 2 includes 175 individuals (132(75.4%) AFAB and 43(24.6%) AMAB) with a mean age equal to 15.9 (± 1.1) years. The distribution of genders assigned at birth significantly differs between clusters 1 and 2 (Chi2, *p* < 0.001).

### Psychiatric history information

Regarding psychiatric history, we found no difference for most variables, including history of psychiatric hospitalizations and school bullying (Table 2). Notably, a large proportion of the participants were hospitalized in a psychiatric ward at least once (*n* = 66, 28%) and were bullied before referral (*n* = 92, 38%), mainly in the school context. However, we found a significantly greater number of schooling breaks (lasting more than 3 months) in the AFAB group than in the AMAB group (*n* = 62 (38.3%) vs. *n* = 16 (21.6%), *p* = 0.012). We also found that the AFAB group had significantly more suicide attempts (*n* = 47 (29%) vs. *n* = 10 (13.5%), *p* = 0.01) and suicidal ideation (*n* = 87 (53.7%) vs. *n* = 22 (29.7%), *p* = 0.001) than the AMAB group. Unfortunately, one individual who was AFAB (trans male) committed suicide during his assessment.

**Table 2:** Psychiatric history and comorbidities of the individuals who were AFAB and AMAB

Variables	AFAB (*n* = 162)	AMAB (*n* = 74)	Total (*n* = 236)	Test	*p* value
*From chart or patient and family interview*					
History of psychiatric hospitalization before consultation	50 (30.9%)	16 (21.6%)	236	Chi2	0.142
History of school bullying before consultation	59 (36.4%)	32 (43.2%)	236	Chi2	0.318
History of school dropout (> 3 months) before consultation	62 (38.3%)	16 (21.6%)	236	Chi2	**0.012**
History of suicidal ideation	87 (53.7%)	22 (29.7%)	236	Chi2	**0.001**
History of suicide attempts	47 (29%)	10 (13.5%)	236	Chi2	**0.01**
*From MINI interview*					
Major depressive disorder	106 (65.4%)	36 (48.6%)	143 (60%)	Chi2	**0.015**
Bipolar disorder	1 (0.6%)	0	2	F	1
Panic disorder	21 (13%)	8 (10.8%)	29 (12%)	Chi2	0.64
Agoraphobia	24 (14.8%)	8 (10.8%)	33 (14%)	Chi2	0.405
Separation anxiety disorder	36 (22.2%)	14 (18.9)	51 (21%)	Chi2	0.565
Generalized anxiety disorder/social anxiety disorder	74 (45.7%)	21 (28.4%)	97 (41%)	Chi2	**0.012**
Phobias	9 (5.6%)	3 (4.1%)	12 (5%)	F	0.758
OCD	5 (3.1%)	3(4.1%)	8 (3%)	F	0.708
PTSD	16 (9.9%)	9 (12.2%)	25 (10%)	Chi2	0.597
Substance use disorder	9 (5.6%)	2 (2.7%)	11 (5%)	F	0.51
Tourette			0		
ADHD	8 (4.9%)	6 (8.1%)	15 (6%)	F	0.378
Conduct disorder	4 (2.5%)	3 (4.1%)	7 (3%)	F	0.681
Oppositional defiant disorder	3 (1.9%)	3 (4.1%)	6 (3%)	F	0.381
Psychotic disorders	10 (6.2%)	3 (4.1%)	13 (5%)	F	0.759
Anorexia nervosa	11 (6.8%)	5 (6.8%)	16 (7%)	Chi2	0.992
Bulimia nervosa	8 (4.9%)	3 (4.1%)	11 (5%)	F	1
Adjustment disorders	31 (19.1%)	11 (14.9%)	42 (18%)	Chi2	0.426
ASD	12 (7.4%)	10 (13.5%)	22 (9%)	Chi2	0.134
Borderline personality disorder	15 (9.3%)	8 (10.8%)	23 (10%)	Chi2	0.709
*Others*					
Learning disability	29 (17.9%)	20 (27%)	50 (21%)	Chi2	0.109

*OCD* obsessive-compulsive disorder, *PTSD* posttraumatic stress disorder, *ADHD* attention deficit hyperactivity disorder, *ASD* autism spectrum disorder. In bold: *p* <  0.05

In terms of psychiatric comorbidities, major depressive disorder was the most frequent psychiatric condition in both groups, but its occurrence was significantly greater in the AFAB group than the AMAB group (*n* = 106 (65.4%) vs. *n* = 36 (48.6%), *p* = 0.015). Similarly, generalized anxiety disorder/social anxiety disorder was more common in the AFAB group (*n* = 74 (45.7%) vs. *n* = 21 (28.4%), *p* = 0.012). Other common psychiatric comorbidities included learning disabilities and separation anxiety. Twenty-two (9%) individuals had ASD and there were no other significant differences in the other diagnoses between the two groups (Table 2).

### Social and medical transitions

By June 2022, 178 (74%) youths had chosen to transition socially within their family, and 144 (61%) had chosen to transition at school. Notably, 96 (40%) youths had already made this decision before their first visit to our clinic. In our group of individuals under 18 years of age, there were significantly more individuals who had transitioned socially both at school and within their families and peer groups in the AFAB group than in the AMAB group. We did not observe significant differences in other aspects of social transition. Specifically, when comparing the age at which social transition occurred at school, within the family, or on civil records, there was no significant difference between the two groups (as indicated in Table 3). The mean age at the beginning of the social transition was 15.13 (± 2.06) years.

**Table 3:** Social and medical transitions of individuals who were AFAB and AMAB

Variables	AFAB (*n* = 162)	AMAB (*n* = 74)	Total (*n* = 236)	Test	*p* value
* Social transition*					
Social transition before the first consultation	71 (43.8%)	23 (31.1%)	96 (40%)	Chi2	0.064
Social transition within the family (before or during care)	128 (79%)	47 (63.5%)	178 (74%)	Chi2	**0.012**
Social transition at school (before or during care)	105 (64.8%)	36 (49.3%)	144 (61%)	Chi2	**0.025**
Change legal name on civil status documents (before or during care)	61 (37.7%)	20 (27%)	83 (35%)	Chi2	0.111
Change gender markers on civil status documents (before or during care)	15 (9.3%)	4 (5.4%)	19 (8%)	Chi2	0.313
Age at the time of social transition at school mean (SD) median [q1; q3]	15.28 (2.29) 15.60 [14.4;16.7]	14.66 (3.43) 15.33 [11.8;17.0]	15,13 (2,6) [4.99-21,42] for *n* = 142	W	0.45
Age at legal name change mean (SD) median [q1; q3]	16.44 (1.6) 16.62 [15.5;17.3]	15.88 (3.04) 16.54 [15.7;17.2]	16,26 (2.06) [8.87-21,17] for *n* = 81	W	0.636
Age at legal gender marker change mean (SD) median [q1; q3]	19.59 (1.04) 19.16 [18.9;19.8]	19.12 (1.64) 18.91 [18.0;20.1]	19,48 (1,17) [17,49 − 21,55] for *n* = 19	W	0.477
*Puberty blockers (GnRH agonists)*					
Number of patients treated with puberty blockers	11 (6.8%)	14 (18.9%)	26 (11%)	Chi 2	**0.005**
Time *(years)* between first consultation and treatment mean (SD) median [q1; q3]	0.61 (0.48) 0.53 [0.25;0.81]	1.04 (1.36) 0.82 [0.28;1.2]	0.84 (1.05) [-1.25-4.63]	W	0.403
Age *(years)* at initiation mean (SD) median [q1; q3]	13.39 (2.65) 13.03 [11.28;14.48]	14.03(1.98) 13.91 [12.36;15.59]	13,87 (2,31) [10.03–17.77]	W	0.467
*Gender-affirming hormones (testosterone or estradiol)*					
Number of patients treated with GAH	76 (46.9%)	28 (37.8%)	105 (44%)	Chi 2	0.193
Time *(years)* between first consultation and treatment mean (SD) median [q1; q3]	1.15 (0.83) 0.9 [0.65;1.29]	1.34 (1.03) 1.18 [0.8;1.39]	1,20 (0,88) [-0.11-5.02]	W	0.221
Age *(years)* at initiation mean (SD) median [q1; q3]	16.93 (1.43) 16.99 [15.77;17.93]	16.73 (1.33) 17.05 [15.82;17.8]	16,87 (1,40) [13.27–20.75]	W	0.712
*Fertility preservation*					
Number of fertility preservation procedures performed	6 (3.7%)	5 (6.8%)	9 (8,57%)	F	0.327

*GAH* Gender-affirming hormone. In bold: *p* <  0.05

Medical interventions can consist of puberty blockers, gender-affirming hormones, fertility preservation, and gender-affirming surgery. Regarding hormonal treatment, 26 (11%) patients received puberty blockers at a mean age of 13.87 (± 2.31) years, and 105 (44%) patients received gender-affirming hormones at a mean age of 16.87 (± 1.4) years. There were no significant differences in the use of gender-affirming hormones or fertility preservation between the two groups (Table 3). However, we found that young trans women (AMAB group) were more likely to use puberty blockers than young trans men (14 (18.9%) vs. 11 (6.8%), *p* = 0.005).

Regarding surgical treatments, 30 (20%) individuals who were AFAB among the 149 individuals who were AFAB aged 12 years or older at their first visit underwent torsoplasty, typically after reaching legal adulthood. The average age for this procedure was 18.44 (± 1.17) years, with the youngest patient being aged 16.03 years and the oldest being aged 21.46 years. Concerning genital surgery, 5 young adults who were AFAB opted for a hysterectomy/oophorectomy, with an average age of 18.8 (± 0.82) years. Metoidioplasty was chosen by only one person and was performed at 20.6 years of age. For feminization surgeries, among the 46 patients aged 12 years and older at the first referral who were AMAB, one young trans female adult underwent vaginoplasty, three underwent facial feminization surgery, and one underwent breast augmentation (mean age 20.5 (± 2.17) years).

## Discussion

Our study examined data from the largest clinic in France for young people with gender issues over 10 years. In this article, we provided specific information on sociodemographic and clinical differences between the AFAB and AMAB groups.

First, we found a greater number of individuals who were AFAB than AMAB, with a ratio of approximately 2/3 individuals who were AFAB for 1/3 individuals who were AMAB. This demographic trend and ratio are similar to those found in other gender clinics around the world since the early 2010s [8]. The reasons for this shift remain unknown. One hypothesis put forward in the literature is the greater stigma attached to trans women than to trans men in society today. Because of this difference in sociocultural acceptance, it could be more difficult for young individuals who are AMAB to seek care or begin transitions [15, 37]. Another reason for the shift in the AFAB/AMAB ratio is that for assigned females, pressure to rapidly conform to feminine gender norms may contribute to the urgent need for clinical services at puberty [38]. In addition, young girls start puberty earlier than young boys and are therefore exposed to gender incongruence phenomena at a younger age due to the development of secondary sexual characteristics [15]. This last hypothesis contradicts the fact that individuals who were AMAB visited the GIC at significantly younger ages than did individuals who were AFAB. Indeed, individuals who were AMAB seemed to be more likely to suffer from prepubertal gender incongruence. This result is congruent with those found in some gender clinics (the Netherlands and Switzerland) but not with those found in others (the UK and Belgium) [14]. Specifically on this last hypothesis, we believe that prepubertal individuals who seek care should be distinguished from pubertal ones. Besides our admixture analysis that supports it, other arguments exist in the literature such as the large multisite study including four Northern countries that reported the shift in AFAB/AMAB ratio only in adolescents [39]. In summary, the role of puberty in the peak number of individuals who were AFAB and sought care in adolescence may be involved, but future studies are necessary to explain this phenomenon and include social hypotheses that are beyond the scope of our retrospective study.

Second, we found no significant difference in social background between the AFAB and AMAB groups in terms of family structure, socioeconomic status, or education. Most children and adolescents in both groups lived with both parents, belonged to middle or high socioeconomic classes, and attended regular schools. Evaluation of these social factors in the literature is very rare in minor trans populations, but data from the Amsterdam Cohort [40] indicate the same family profile. Among adults, however, several studies indicate that trans people may experience growth in more disadvantaged and marginalized environments [41, 44]. Additionally, a recent large epidemiological study in the UK revealed that the prevalence of trans identity was correlated with the level of socioeconomic deprivation [5]. Because our patient group consisted exclusively of children and adolescents, they had not yet integrated into working life and were therefore possibly less exposed to the socioeconomic consequences of discrimination experienced by the adult trans population. Another possible bias regards access to care in special clinics, which may be more difficult for youths in poor socioeconomic contexts favoring middle or high socioeconomic classes.

Third, as we expected, transgender youths face significant mental health challenges and social exclusion. In particular, we specifically found evidence that young individuals who were AFAB exhibited more severe mental health issues than those who were AMAB: there were significant differences in terms of suicidality (more suicide attempts, more suicidal ideation), as well as a greater incidence of psychiatric comorbidities, particularly major depressive disorders, and anxiety disorders. These findings align with prior research [25, 26] and may offer additional insight into why a greater proportion of individuals who are AFAB seek treatment at gender clinics. On the other hand, fewer studies have reported different results, with AMAB individuals experiencing more mental health problems [28, 29]. Overall, more individuals who were AFAB than individuals who were AMAB met the criteria for gender incongruence (from the ICD-11), which could partly explain their greater distress. This greater rate of gender incongruence could again be explained by the impact of puberty, which occurs earlier in individuals who are AFAB. Previous studies have conducted a comprehensive analysis of the distress experienced by transgender youth and have identified two primary types of stress. As explained by Wittlin et al. in 2023, “externalized stressors” include discrimination, victimization, and rejection, and “internalized stressors” include the anticipation of discrimination, concealment of aspects of one’s identity, and the internalization of stigma. Recent discussions in the literature suggest that AFAB individuals may encounter more externalized problems, while AMAB individuals may experience more internalized issues [14]. Our study did not find a disparity in school bullying rates; however, we noted a greater occurrence of school dropout among AFAB individuals. We did not specifically study peer relationships. Therefore, further research is needed to elucidate the causal connections between these distinct stressors and their impacts on mental health, which seems to be more adversely impacted in individuals who are AFAB.

Finally, many of these young people sought help from our clinic to support them in their gender transitions. Regarding transition paths, our results indicate that more AMAB youth choose to transition socially (within their families and at school) than youth who are AMAB. However, among those who chose to transition socially (i.e., the majority of the patients in our cohort), there was no age difference between the two groups. This might indicate that individuals who are AMAB are more likely to “hide” their trans identity at a young age due to the strong stigma attached to trans women and other factors yet to be studied and to transition later. Studies on the social transitions of adolescents are still rare. In our cohort, a smaller proportion of youth opted to transition through medical intervention with hormone treatments: 11% received puberty blockers, while 44% received gender-affirming hormone therapies. These percentages are notably lower than those reported in the Amsterdam cohort [30] which is currently a significant source of literature on this topic. This difference might be attributed to variations in societal acceptance, with trans identity and gender transition being more widely accepted and recognized in Northern European countries. Numerous recent publications, as well as the recommendations of the WPATH, support the short-term psychosocial benefits of these hormone treatments for young people who request them [42, 43]. In our cohort, a significantly greater number of individuals who were AMAB than individuals who were AFAB received puberty blockers. This finding diverges from previous research reported in the literature [30, 44]. However, this statistical variance could be due to the relatively low total number of patients who opted to receive puberty blockers in our study. Future observations of our cohort will examine how these patterns evolve, especially as the patient population at the Gender Identity Clinic (GIC) grows annually. No other significant differences were noted regarding the use of gender-affirming hormones or the pursuit of fertility preservation options. Finally, the most frequently performed surgical treatment was torsoplasty for individuals who were AFAB. Because of the small number of young people who had undergone surgical treatment, we did not perform any statistical comparisons between the two groups. Surgical treatments, which require the agreement of legal guardians, are still rare among minors.

Our study has certain limitations. Although the GIC helps the largest number of young transgender children and adolescents in France, data is only available for 239 people from a single center. Additionally, the cohort appears to be largely from a uniform social class, even though the patients accessed our services in various ways (such as referrals by doctors, associations, or on their own). Due to these factors, our findings may not be generalizable to the entire population of transgender youth. Moreover, in response to recent shifts within this population, particularly the documented increase in the number of individuals who are AFAB, we wanted to provide the most recent data possible (June 2012 to March 2022). Future research with an extended period of follow-up will be necessary to further investigate this population, which seems to increase every year.

In conclusion, age at referral should be considered when exploring gender incongruence as shown by the change in sex ratio in the two clusters from the admixture analysis. Our study also revealed significant differences between young transgender individuals who were assigned female or male at birth, especially in sociodemographic and clinical aspects, as well as in their gender transition pathways. This study provides us with important additional information for understanding this increasingly numerous and vulnerable population at the heart of real societal change. Further research to fundamentally understand the existing differences between these two groups, and how we might adjust our care according to the vulnerabilities and needs specific to each individual according to their sex assigned at birth and the gender expressed is needed.

## Data Availability

The data presented in this study are available on appropriate request from the corresponding author. The data are not publicly available as the privacy of the human subjects must be ensured.
